# Correction: The relationship between screen time, screen content for children aged 1–3, and the risk of ADHD in preschools

**DOI:** 10.1371/journal.pone.0341722

**Published:** 2026-01-23

**Authors:** Jian-Bo Wu, Yanni Yang, Qiang Zhou, Jiemin Li, Wei-Kang Yang, Xiaona Yin, Shuang-Yan Qiu, Jingyu Zhang, Minghui Meng, Yawei Guo, Jian-hui Chen, Zhaodi Chen

The authors, Jian-hui Chen and Zhaodi Chen, should be noted as shared co-corresponding authors on this work. Zhaodi Chen’s email address is: 13430268460@163.com.
