# Supplementary material for: The relationship between screen time, screen content for children aged 1-3, and the risk of ADHD in preschools
Source: PLoS One. 2025 Apr 23;20(4):e0312654. doi: 10.1371/journal.pone.0312654 (PMC12017831; doi:10.1371/journal.pone.0312654)
Supplement: Attachment — Submitted filename: Comments and feedback.pdf [file pone.0312654.s002.pdf]

General Feedback:

The study deals with an important current issue concerning the children's amount of time spent on screens and its probable association with ADHD. It uses a good (robust) sample size, which strengthens its results considerably. However, certain parts may need further clarification and elaboration to strengthen the manuscript.

Specific Comments:

1. The title effectively summarizes the study's focus; however, including the specific age group studied would add further clarity.
2. The abstract is well-constructed, briefly mention the key limitations, such as the cross-sectional design.
3. The introduction is thorough, but the transition from general concerns about screen time to specific research gaps feels abrupt. Consider expanding on these gaps to create a smoother flow.
4. Citations should be more seamlessly integrated to strengthen statements about existing evidence and research gaps.
5. While the description of participant sampling is clear, it would be helpful to specify whether recruitment was conducted through randomization or convenience sampling from kindergartens.
6. Ethical considerations are addressed; however, the rationale behind the age group selection for screen exposure (1–3 years) and ADHD assessment (4–7 years) should be provided.
7. The use of the SDQ is appropriate, but the justification for the ADHD subscale cut-offs used in this population, along with information on whether cultural adaptations of the SDQ were validated, should be included.
8. Provide further explanation for categorizing interactive videos separately, including how these were defined for parents completing the questionnaire.

Statistical Analysis:

9. Logistic regression is appropriate for this study, but more details about model diagnostics (e.g., multicollinearity checks, model fit) would enhance the rigor.
10. Explain if any interaction terms between covariates were explored.

Results:

Data Presentation:

11. The tables are informative but could be improved for clarity. For example, in Table 2, the heading "ADHD Risk (%)" is somewhat misleading, as it presents the percentage of cases in each category rather than the actual risk of ADHD or percentage risk. It would be more appropriate to label this column as "No. (%)" and include the term “ADHD risk” specifically in rows labeled "Edge value" and "Abnormal."
12. Highlight inconsistencies or unexpected trends in results (e.g., the non-significance of interactive videos) and discuss possible reasons.
13. The interpretation of odds ratios is clear, but confidence intervals for non-significant results should also be discussed to avoid misinterpretation.
14. Consider including a full model table as supplementary material, incorporating all confounding variables. This would allow readers to better understand the effect of screen time after adjusting for age, gender, and other confounding factors. Additionally, it would be insightful to observe whether any confounding variables still show a significant association with the risk of ADHD after adjustment. Also add the information about goodness of fit and classification table.

Link to Literature:

14. The discussion contextualizes findings well but could include more international studies to generalize the findings beyond China.

Strengths and Limitations:

15. The limitations are acknowledged but could be expanded. For example, discuss potential biases from self-reported screen time and ADHD symptoms.

Policy Implications:

16. The recommendations for managing screen time are practical but generic. Provide examples of actionable strategies for parents and educators.
17. The conclusion is concise but could briefly outline future research directions, such as longitudinal studies or intervention trials.

Queries for Authors:

1. Was the order of covariate inclusion in the logistic model based on theoretical considerations or empirical testing?
2. Were there differences in ADHD risk between rural and urban settings within the district?
3. How was parental awareness or understanding of ADHD assessed in this study?

Suggestions for Improvement:

4. Enhance the clarity of key concepts in the introduction, particularly regarding how different screen contents might influence ADHD risks differently.
5. Improve the visualization of results, possibly through graphical summaries like forest plots for odds ratios.

This study has the potential to inform public health policies on screen time management, but addressing the above points will make its contributions even more compelling.
