# Supplementary material for: The relationship between screen time, screen content for children aged 1-3, and the risk of ADHD in preschools
Source: PLoS One. 2025 Apr 23;20(4):e0312654. doi: 10.1371/journal.pone.0312654 (PMC12017831; doi:10.1371/journal.pone.0312654)
Supplement: Attachment — Submitted filename: Comments on Revision-1.pdf [file pone.0312654.s004.pdf]

Subject: Review of Revised Manuscript – Final Feedback and Refinements

Dear Zhaodi Chen,

Thank you for submitting the revised version of your manuscript, The Relationship Between Screen Time, Screen Content for Children Aged 1-3, and the Risk of ADHD in Preschools (Manuscript No: PONE-D-24-42830R1). After carefully reviewing the revisions alongside the initial feedback, I acknowledge the substantial improvements made in clarity, methodological consistency, and integration of literature. Below is my evaluation of how the key concerns were addressed, along with final recommendations for minor refinements.

1. General Improvements

Title & Abstract: The revised title now clearly specifies the study population, improving clarity. The abstract now acknowledges the cross-sectional design as a key limitation.

Introduction Clarity & Research Gaps: The transition from general concerns to specific research gaps has been improved, strengthening the study's rationale.

Citations & Literature Integration: Citations are now more seamlessly incorporated, providing stronger support for the study's claims.

2. Methodological Clarity & Ethical Considerations

Participant Recruitment: The clarification that data were collected from the Longhua Child Cohort Study (LCCS) and that all kindergartens in the district were included effectively addresses concerns about sampling.

Justification of Age Groups: The revised manuscript now provides a developmental rationale for assessing screen exposure at 1-3 years and ADHD symptoms at 4-7 years.

Validation of ADHD Measure (SDQ): The explanation that SDQ norms for the Chinese population have been established and validated adds credibility to the ADHD risk assessment.

3. Statistical Analysis Enhancements

Multicollinearity & Model Fit: The inclusion of multicollinearity checks and Hosmer-Lemeshow test results ensures statistical robustness.

Inclusion of Confounding Variables: The addition of a full model table in the supplementary materials strengthens the study's methodological transparency.

Interaction Terms: While not explored in this study, their potential impact has been acknowledged as a direction for future research.

4. Results Presentation & Interpretation

Table Refinements: Table 2 has been revised for clarity, replacing "ADHD Risk (%)" with "No. (%)" to prevent misinterpretation.

Unexpected Findings (Interactive Videos): The non-significant effect of interactive videos is now bolded in the results and further discussed.

Confidence Intervals: CI ranges for non-significant findings have been addressed to avoid misinterpretation.

Visualization of Results: A forest plot for odds ratios has been added, improving data presentation.

5. Discussion & Literature Integration

International Comparisons: More references to international studies have been included, improving generalizability.

Limitations Expanded: The study now acknowledges recall bias, self-reporting limitations, and unmeasured confounders.

Policy Implications: Practical recommendations for parents and educators have been incorporated, such as setting screen time limits and encouraging breaks.

Future Research Directions: The conclusion now highlights the importance of longitudinal studies and intervention trials.

6. Response to Reviewer-Specific Queries

Covariate Selection: It is now clarified that covariates were included based on both theoretical and empirical considerations.

Rural vs. Urban ADHD Risk Differences: The study focuses on an urban population, with a note that future research should explore rural-urban variations.

Parental Awareness of ADHD: While not directly assessed, this has been acknowledged as a limitation.

7. Final Recommendations for Minor Refinements

While the manuscript has been significantly strengthened, I recommend incorporating the following minor refinement:

Explicitly acknowledge potential selection bias due to parental self-reporting, particularly regarding screen time and ADHD symptoms.

Suggested Placement: This can be added in the Limitations section, expanding on the discussion of recall bias.

Suggested Wording: "Another limitation of this study is the reliance on parental self-reports for both screen time and ADHD symptoms. Self-reported data may introduce bias due to social desirability or inaccurate recall. Future studies should consider incorporating objective measures, such as device tracking for screen time and clinical assessments for ADHD diagnosis, to enhance data reliability."

Overall, this revision demonstrates substantial improvement in clarity, methodology, and policy relevance. I appreciate the authors' thorough responses to the feedback and believe the manuscript is now in strong shape for acceptance.

Best regards,

Dr. Muhammad Aasim
