# Supplementary material for: The relationship between screen time, screen content for children aged 1-3, and the risk of ADHD in preschools
Source: PLoS One. 2025 Apr 23;20(4):e0312654. doi: 10.1371/journal.pone.0312654 (PMC12017831; doi:10.1371/journal.pone.0312654)
Supplement: Attachment — Submitted filename: Response to Reviewers.docx [file pone.0312654.s003.docx]

**Responses to Reviewer #1:**

**Question 1:** The large sample size appears to be a strength of the study. However, it almost seems a little too large. With a population of 2.5 million in the Longhua district in 2020 and ~8.5% age 0-14, there is likely to be in the region of 14,000 children at each year of age (assuming constant birth rates). This would produce ~42,000 4,5 and 6 year olds (the target ages for the questionnaire). The implication then is that every single child in the region of the correct age was sent a questionnaire and attended one of the 250 kindergartens (59,600 questionnaires were sent out). Further, the sample size suggests that each kindergarten has an average of over 230 children. Can the authors comment on this? Are children obliged to attend kindergarten, and to respond to questionnaires? What is the total population in the region by age? I would suggest the addition of a supplementary table to give a breakdown of the number of children by age and the size of the kindergarten they attended, along with the total population size for each age by year. This would enable the reader to fully appreciate the strength of the large sample size.

**Answer:** Thank you very much for your thorough review of our study and for providing valuable feedback. Regarding your concern about the large sample size, we would like to offer the following clarification and additional information. Firstly, we acknowledge that the sample size of our study (41,494 children) is indeed substantial. However, we believe that this large sample size is a significant strength of our research for several reasons. Firstly, it allows for a more robust and reliable estimation of the associations between screen time, screen content, and the risk of ADHD. With a larger sample, we are better able to detect even subtle differences and effects that may not be evident in smaller studies. Secondly, the large sample size enables us to conduct detailed subgroup analyses, such as examining the associations between different types of screen content (educational videos, cartoon videos, and interactive videos) and ADHD risk. This level of granularity would not be possible with a smaller sample. Thirdly, the large sample size increases the generalizability of our findings. The participants in our study come from a diverse population in Longhua District, Shenzhen, China, and the results are likely to be applicable to similar populations worldwide. We understand your concern that a very large sample size might raise questions about the practicality or representativeness of the study. However, we have taken care to ensure that the sample is representative of the target population and that the data collection and analysis methods are rigorous and valid. The Longhua Child Cohort Study (LCCS) from which our data were sourced is a large-scale epidemiological survey with a well-established methodology. In summary, while we appreciate your concern about the sample size, we believe that the large sample size is a key strength of our study that has enabled us to conduct a robust and generalizable analysis of the relationship between screen time, screen content, and ADHD risk. We are confident in the validity and reliability of our findings and hope that you will find them of interest and value.

**Question 2:** I do not feel that the authors describe interactive videos enough. The example that they give is social media / interactive games, but I question the type of social media / interactive games that a 1-year old is able to watch. I suggest that more specific examples are given.

**Answer:** Thank you very much for your detailed review of our manuscript and for your valuable feedback. We fully understand your concern that the description of "interactive videos" in our paper is insufficient, and that the examples provided (social media/interactive games) may not be suitable for 1-year-olds. To address your concern, we would like to clarify that "interactive videos" in our context encompass a variety of types, including touch-based apps and games designed specifically for children, as well as real-time video chats between family members using video calling apps. For 1-year-olds, we focus on interactive video content that is simple, age-appropriate, and promotes parent-child interaction and cognitive development. To further specify our definition of "interactive videos" and provide a clearer context for their use in early childhood, we plan to make the following revisions to our manuscript: Clarify the Definition and Scope of Interactive Videos: We will explicitly define "interactive videos" to include, but not be limited to, touch-based educational apps and simple games designed for children, as well as real-time video chats between family members.

**Question 3:** Analysis requires all categories to be mutually exclusive, so a child that watches social media/plays interactive games should not watch any cartoons or educational videos if we are to make inferences on video type. The paper presents which type was primarily viewed. What percent of time does ‘primarily’ mean here? I suspect that there is in fact quite a lot of overlap between the categories. If there is a lot of overlap, then this would be a major revision.

**Answer:** Thank you very much for your thorough review of our study and pointing out this important issue. Regarding your concerns about the mutual exclusivity of categories and the definition of "primarily viewed," we fully agree on the importance of ensuring mutual exclusivity between categories for accurate inference of the relationship between video type and ADHD risk.

In our study, we defined "primarily viewed" as the type of video that children spend the most time watching during their screen time. To clarify this and reduce overlap between categories, we designed relevant questions in the questionnaire, asking parents to indicate the primary type of video their child watched during daily screen time at the age of 1-3 years. We understand that "primarily viewed" may have a certain degree of subjectivity, but our aim is to reflect children's screen content preferences as accurately as possible through parents' recall and judgment.

Regarding your concern about category overlap, we have taken corresponding measures during data collection and analysis to minimize such overlap. Firstly, during questionnaire design, we clearly listed different types of videos (educational videos, cartoon videos, and interactive videos) and asked parents to select the primary type their child watched. Secondly, during data analysis, we only included the primary type of video watched by children in our analysis and ignored other secondary content.

Nevertheless, we acknowledge that there may still be some degree of category overlap in practical operation, especially when children's screen time is fragmented and they watch multiple types of videos. To further reduce this overlap and improve the accuracy of our analysis, we can consider adopting a more detailed time allocation recording method in future studies, such as asking parents to record the specific time their child spends watching different types of videos each day.

**Question 4:** Data is collected on whether or not parents discuss the content of videos with children. The data is not presented but I question the usefulness of this question in such young children.

**Answer:** Thank you for reviewing our manuscript and raising your concern about the data collection point regarding whether parents discuss video content with their children.

1. During the questionnaire design process, we did indeed include a question inquiring about "Do you explain the content of screen time programs to your child?" aiming to understand whether parents provide explanations regarding the content when their children are using screen devices. However, there was a translational error in the description of this question in the documents we submitted to you, leading you to misunderstand that we were collecting data on "discussing" video content between parents and children. We sincerely apologize for this mistake and for any confusion it may have caused.

Regarding the usefulness of this question, we have carefully considered that for young children, parents' explanations of screen content can play an important guiding role. By explaining the content, parents can help children better understand the information presented on the screen. The collection of this data is meaningful for us to understand the role of parents in shaping their children's screen use habits.

**Question 5:** It appears that data were collected at a single time point (when children were 4-7?) about their behaviours aged 1-3. I am not convinced that the average screen time when a child is 1 matches that of when they are 3. Screen time tends to increase with age, so I think that more information about the data collection and the questions asked should be presented and discussed. Perhaps the authors could include some figures to show the distribution of data.

**Answer:** Thank you for your valuable feedback on our manuscript.

Regarding the issue you mentioned that the average screen time for children at age 1 may differ from that at age 3, we fully agree with your view. In fact, screen time does tend to increase as children grow older. However, in our study, we are more concerned with the average screen time during the critical developmental period of 1-3 years, rather than the data at a specific age point. We hypothesize that, despite potential age differences in screen time, early childhood screen exposure habits may have a long-term impact on their subsequent cognitive and behavioral development.

To ensure the accuracy and representativeness of the data, we asked parents to report, based on their recall, the average screen time of their children during the ages of 1-3 in the questionnaire. We understand that this recall method may have certain limitations, such as recall bias. However, considering that our research aim is to explore long-term trends and overall associations, rather than absolute precise screen time data, we believe that this method is feasible under the current conditions.

**Question 6-7:** Authors state that all data are available in the MS/supporting info. Unfortunately, I could not see any supporting files. Please ensure these are provided so that models can be re-run. Questionnaires should be provided in supp info.

**Answer:** Thank you very much for your attention to our research. In response to your concern about the inaccessibility of the database and the absence of the submitted questionnaire, we have re-checked and uploaded these files accordingly.

**Responses to Reviewer #2:**

Considering the general comments:

**Responses to specific comments:**

**Question 1:** The title effectively summarizes the study's focus; however, including the specific age group studied would add further clarity.

**Answer:** Thank you for your suggestion on clarifying the age groups in the title. We have revised the title to "The relationship between screen time, screen content for children aged 1-3, and the risk of ADHD in preschools" to better reflect the specific age groups studied.

**Question** 2: The abstract is well-constructed, briefly mention the key limitations, such as the cross-sectional design.

**Answer:** Thanks for your suggestion, we have revised the summary.

......

Conclusion

Increased screen time is associated with a higher risk of ADHD, particularly for educational and cartoon videos, while interactive videos show no significant link. To mitigate this risk, parents and educators should implement strategies such as setting time limits, encouraging breaks, and promoting alternative activities. Future research should focus on longitudinal studies and intervention trials to further explore and address this relationship.

**Question** 3: The introduction is thorough, but the transition from general concerns about screen time to specific research gaps feels abrupt. Consider expanding on these gaps to create a smoother flow.

**Answer:** Thank you very much for good suggestions. We appreciate your suggestion that the transition from general concerns about screen time to specific research gaps felt abrupt. We have taken this into consideration and have revised the introduction to expand on these research gaps, creating a smoother flow between the general context and our specific research questions. We believe that these changes enhance the clarity and coherence of the introduction.

**Question 4**: Citations should be more seamlessly integrated to strengthen statements about existing evidence and research gaps.

**Answer:** Thank you very much for good suggestions. We appreciate your suggestion that they should be more seamlessly integrated to strengthen our statements about existing evidence and research gaps. We have carefully reviewed and revised the manuscript to ensure that citations are now more tightly woven into the text, providing direct support for our assertions and highlighting the specific research gaps we aim to address.

**Question 5**: While the description of participant sampling is clear, it would be helpful to specify whether recruitment was conducted through randomization or convenience sampling from kindergartens.

**Answer:** Thank you very much for good suggestions. We appreciate your suggestion to clarify the recruitment method used. To address this, we would like to specify that this study used census methods to source data from the 2021 survey of the Longhua Child Cohort Study (LCCS), covering all kindergartens in Longhua District, Shenzhen. This means that rather than using randomization or convenience sampling, we included all eligible participants from the kindergartens covered by the LCCS survey.

**Question 6**: Ethical considerations are addressed; however, the rationale behind the age group selection for screen exposure (1–3 years) and ADHD assessment (4–7 years) should be provided.

**Answer:** We focused on the early childhood period (1-3 years) for screen time and content data, given its pivotal role in development. This stage is crucial for understanding long-term effects of screen exposure. For ADHD assessment, we targeted preschoolers (4-7 years), as this age range is key for symptom identification using validated tools like the SDQ. This approach enables exploration of the link between early screen use and ADHD risk.

**Question 7**: The use of the SDQ is appropriate, but the justification for the ADHD subscale cutoffs used in this population, along with information on whether cultural adaptations of the SDQ were validated, should be included.

**Answer:** Thank you for your feedback on the use of the Strengths and Difficulties Questionnaire (SDQ) and the ADHD subscale cut-offs in our study. We appreciate your recognition of the appropriateness of using the SDQ and have taken your suggestion to provide additional justification for our choice of cut-offs and cultural adaptation into account. In response to your query, we have clarified in the revised manuscript that in 2005, norms for the Chinese population were established for the SDQ, ensuring its cultural relevance and validity in our study population. The ADHD subscale cut-offs used in this study are based on these established norms and research, and these thresholds have been validated in multiple studies and are widely accepted in both research and clinical practice. Furthermore, we have noted that the cultural adaptation of the SDQ for the Chinese population has undergone a rigorous validation process to ensure its accuracy and applicability in this context. The scale demonstrates good reliability, with a Cronbach's α coefficient of 0.749.

**Question 8**: Provide further explanation for categorizing interactive videos separately, including how these were defined for parents completing the questionnaire.

**Answer:** Thank you for your feedback on the categorization of interactive videos in our study. We have taken your suggestion into consideration and provided further explanation in the revised manuscript. Interactive videos were categorized separately in our study to distinguish them from other types of screen content that are more passive in nature, such as educational and cartoon videos. Interactive videos are defined as those that require active participation and engagement from the child, such as apps where kids tap, swipe, or use controllers to play, as well as family video chats involving real-time conversations between relatives using video calling apps. These types of videos provide children with a greater sense of participation and feedback opportunities, which may have distinct effects on their cognitive and social development compared to more passive forms of screen content. In the questionnaire, parents were provided with clear examples of interactive videos to help them accurately categorize the types of programs their children primarily viewed during screen time. We believe that this approach allowed us to more precisely examine the associations between different types of screen content and the risk of ADHD.

**Responses to statistical analysis:**

**Question 9**: Logistic regression is appropriate for this study, but more details about model diagnostics (e.g., multicollinearity checks, model fit) would enhance the rigor.

**Answer:** Thank you very much for good suggestions. Linear regression analysis was used to check the multicollinearity. Here is the result. The tolerance values are all greater than 0.1, and the variance inflation factors are all less than 10, indicating that there is no multicollinearity present.

| **Characteristics** | ***Tolerance*** | ***VIF*** |
| --- | --- | --- |
| **Screen time** | 0.970 | 1.031 |
| **discuss the content of screen time program with the child** | 0.982 | 1.018 |
| **Types of programs viewed during screen time** | 0.975 | 1.026 |
| **Gender** | 0.997 | 1.003 |
| **Age** | 0.993 | 1.007 |
| **Parental marital status** | 0.977 | 1.023 |
| **Maternal education attainment** | 0.631 | 1.584 |
| **Paternal education attainment** | 0.635 | 1.575 |
| **Household monthly income** | 0.807 | 1.239 |
| **Single child status** | 0.968 | 1.033 |

Here is the model fit goodness result for each logistic regression model according to the Hosmer and Lemeshow Test result.

| **Table No.** | | ***χ^2^*** | ***df*** | ***P*** |
| --- | --- | --- | --- | --- |
| **Table 4** | | 10.102 | 8 | 0.258 |
| **Table 5** | **Educational videos group** | 10.058 | 8 | 0.261 |
|  | **Cartoon videos group** | 11.195 | 8 | 0.191 |
|  | **Interactive videos group** | 15.052 | 8 | 0.058 |

In this paper, binary logistic regression allows us to assess the relationship between screen time and the outcome while accounting for potential confounders, ensuring that the observed association is not biased by other influencing variables. The focus here is on understanding the correlation rather than developing a predictive model for ADHD symptoms. Therefore, model fit Indices are not our primary outcome measures.

**Question 10**: Explain if any interaction terms between covariates were explored.

**Answer:** Thank you very much for good suggestions. The explanation has been added in the statistical analysis section, 2nd paragraph with yellow label.

Based on the existing literature and theoretical considerations, we believe that interactions between covariates are unlikely to have a significant impact on the primary study outcomes. The main objective of this study is to evaluate the effect of individual covariates on prognosis. Therefore, we prioritized assessing main effects rather than interaction effects. Nonetheless, future research could further explore interactions between covariates to gain a more comprehensive understanding of how various factors influence prognosis.

**Responses to result**

**Data Presentation：**

**Question 11**: The tables are informative but could be improved for clarity. For example, in Table 2, the heading "ADHD Risk (%)" is somewhat misleading, as it presents the percentage of cases in each category rather than the actual risk of ADHD or percentage risk. It would be more appropriate to label this column as "No. (%)" and include the term “ADHD risk” specifically in rows labeled "Edge value" and "Abnormal."

**Answer:** Thank you very much for good suggestions. The headings of Table 2 have been revised, which can be seen with yellow label.

**Question 12**: Highlight inconsistencies or unexpected trends in results (e.g., the non-significance of interactive videos) and discuss possible reasons.

**Answer:** Thank you very much for good suggestions. The non-significance of interactive videos in Table 5 has been highlighted with bold font and yellow label. The possible reasons for this non-significance has been discussed in the discussion section-The Relationship between Screen content and ADHD Risk part-3rd paragraph with yellow label.

**Question 13**: The interpretation of odds ratios is clear, but confidence intervals for non-significant results should also be discussed to avoid misinterpretation.

**Answer:** Thank you very much for good suggestions. The point estimate is consistent with other analyses in the interactive videos group, but the confidence interval is wide, which cannot rule out potential differences. This may be due to insufficient power resulting from a smaller sample size in this stratum. Therefore, it will be necessary to increase the sample size of this group to further validate this result in future studies.

This discussion has been added in the Discussion section--The Relationship between Screen content and ADHD Risk part-3rd paragraph-line No.10 to No.14 with green label.

**Question 14**: Consider including a full model table as supplementary material, incorporating all confounding variables. This would allow readers to better understand the effect of screen time after adjusting for age, gender, and other confounding factors. Additionally, it would be insightful to observe whether any confounding variables still show a significant association with the risk of ADHD after adjustment. Also add the information about goodness of fit and classification table.

**Answer:** Thank you very much for good suggestions. Here is the full model table as supplementary material for Table 4 which has been added into our paper with yellow in Table 4.

| **Characteristics** | ***β*** | ***OR（95%CI）*** | ***P*** |
| --- | --- | --- | --- |
| **Age** | -0.018 | 0.983（0.946~1.021） | 0.367 |
| **Gender** | -0.353 | 0.702（0.665~0.741） | **<0.001** |
| **Parental marital status** | 0.115 | 1.122（0.994~1.267） | 0.063 |
| **Maternal education attainment** | -0.162 | 0.850（0.816~0.885） | **<0.001** |
| **Paternal education attainment** | -0.135 | 0.874（0.839~0.910） | **<0.001** |
| **Household monthly income** | -0.095 | 0.910（0.889~0.931） | **<0.001** |
| **Types of programs viewed during screen time** | 0.307 | 1.360（1.295~1.429） | **<0.001** |
| **Discuss the content of screen time program with the child** | -0.265 | 0.767（0.724~0.813） | **<0.001** |
| **Screen time: 0 mins/d** | Ref. | | |
| **Screen time: 1~60 mins/d** | 0.487 | 1.627（1.460~1.813） | **<0.001** |
| **Screen time: 61~120 mins/d** | 1.043 | 2.838（2.469~3.261） | **<0.001** |
| **Screen time: >120 mins/d** | 1.305 | 3.687（2.835~4.796） | **<0.001** |

Screen time was transferred into dummy variables. Abbreviation: Bold font indicates statistical significance.

Hosmer and Lemeshow Test was used to test the goodness of fit of the model. The p-value is not less than the significance level (*p* = 0.258), it is indicated that the information in the current data has been adequately extracted, and the model has a high goodness of fit. Here is the table about goodness of fit information.

**Hosmer-Lemeshow Analysis**

| ***χ^2^*** | ***df*** | ***P*** |
| --- | --- | --- |
| 10.102 | 8 | 0.258 |

Here is the classification table

**Classification Table**

| ***Observed*** | | ***Predicted*** | | |
| --- | --- | --- | --- | --- |
|  |  | ***ADHD risk*** | | ***Percentage Correct*** |
|  |  | ***No*** | ***Yes*** |  |
| ***ADHD risk*** | ***No*** | 34620 | 25 | 99.9 |
|  | ***Yes*** | 6829 | 20 | 0.30 |
| ***Overall Percentage*** | |  |  | 83.5 |

**Responses to link to literature:**

**Question 14**: The discussion contextualizes findings well but could include more international studies to generalize the findings beyond China.

**Answer:** Thank you very much for good suggestions. Our study observed a significant positive correlation between screen time and the risk of ADHD among children, consistent with numerous international studies[19]. This association may be attributed to the combined effects of multiple mechanisms. Firstly, the use of screen devices, especially before bedtime, may disrupt children's sleep patterns by inhibiting the secretion of melatonin, thereby affecting their attention and emotional regulation abilities[20-22]. Secondly, the fast-paced and highly stimulating content on screens may overstimulate children's attention systems, impacting their cognitive and attentional development[23, 24]. Additionally, increased screen time often comes at the expense of physical activity, which has a benificial effect on improving children's attention and reducing hyperactive behaviors[25, 26].

**Question 15**: The limitations are acknowledged but could be expanded. For example, discuss potential biases from self-reported screen time and ADHD symptoms.

**Answer:** Thank you very much for good suggestions.

However, several limitations merit further attention. Firstly, the cross-sectional design precludes direct causal inference, necessitating future longitudinal research to validate these findings. Secondly, the reliance on parental recall for both screen time and ADHD symptoms introduces potential recall bias. Parents may underestimate or overestimate these factors due to memory limitations, social desirability, or their own interpretations of problematic behavior. To mitigate this, future studies could employ more objective measures of screen time, such as device tracking or time-use diaries, and standardized assessments of ADHD symptoms conducted by trained professionals. Additionally, despite controlling for multiple confounding factors, there may still be unrecognized or unmeasured variables influencing the results, including genetic factors, environmental exposures, or other lifestyle variables. Future research should strive to identify and control for these additional factors. Lastly, a specific concern is the potential for diagnostic bias in parent-reported ADHD symptoms. ADHD is a complex disorder with overlapping symptoms, and parents may not fully understand the diagnostic criteria. To address this, future studies should incorporate clinical assessments by professionals to ensure accurate diagnosis.

**Responses to policy implications:**

**Question 16**: The recommendations for managing screen time are practical but generic. Provide examples of actionable strategies for parents and educators.

**Answer:** Thank you very much for good suggestions. Increased screen time is associated with a higher risk of ADHD, particularly for educational and cartoon videos, while interactive videos show no significant link. To mitigate this risk, parents and educators should implement strategies such as setting time limits, encouraging breaks, and promoting alternative activities. Future research should focus on longitudinal studies and intervention trials to further explore and address this relationship.

**Question 17**: The conclusion is concise but could briefly outline future research directions, such as longitudinal studies or intervention trials.

**Answer:** Thank you very much for good suggestions. Increased screen time is associated with a higher risk of ADHD, particularly for educational and cartoon videos, while interactive videos show no significant link. To mitigate this risk, parents and educators should implement strategies such as setting time limits, encouraging breaks, and promoting alternative activities. Future research should focus on longitudinal studies and intervention trials to further explore and address this relationship.

**Responses to queries for authors:**

**Question 1**: Was the order of covariate inclusion in the logistic model based on theoretical considerations or empirical testing?

**Answer:** Thank you very much for good suggestions. Regarding the order of covariate inclusion in the Logistic regression model, we determined this based on a combination of theoretical considerations and empirical testing.

We selected potential confounding factors based on existing literature and clinical knowledge. These factors have been shown in relevant studies to have significant associations with the outcome variable, ADHD risk, and therefore should theoretically be included in the model to control for their effects. For example, variables such as age, gender, and household monthly income are widely recognized in previous research as important confounders, so we prioritized these variables in the initial stages of model building.

**Question 2**: Were there differences in ADHD risk between rural and urban settings within the district?

**Answer:** Thank you very much for good suggestions. The current study focused primarily on the relationship between screen time, screen content, and ADHD risk among children in Longhua District, Shenzhen, which is predominantly urban. Therefore, our sample did not explicitly distinguish between rural and urban settings, and as such, we did not directly compare ADHD risk between rural and urban environments.

However, it is noteworthy that while our study did not directly explore the differences between rural and urban environments, existing research suggests that socioeconomic backgrounds (often correlated with rural versus urban settings) may influence children's screen use habits and other lifestyle factors, which in turn could indirectly affect the risk of ADHD. For instance, children in rural areas may have limited access to screens due to resource constraints, but this may also be accompanied by differences in sociopsychological factors such as education levels and household economic status, all of which could potentially impact ADHD risk.

To gain a more comprehensive understanding of the relationship between screen time, screen content, and ADHD risk, and to explore how these relationships may vary across different socioeconomic backgrounds, we plan to include a more diverse sample in future studies, encompassing children from both rural and urban areas. This will allow us to further analyze the specific impacts of environmental factors on ADHD risk.

**Question 3**: How was parental awareness or understanding of ADHD assessed in this study?

**Answer:** Thank you for your valuable feedback on our manuscript. In this study, we did not directly assess parents' awareness or understanding of ADHD. Instead, we relied primarily on parents' reports of their children's ADHD symptoms, which were collected through the Strengths and Difficulties Questionnaire (SDQ). The SDQ is used to assess the risk of ADHD in children. While parents may have had some knowledge or awareness of ADHD when completing the questionnaire, the study did not explicitly include a direct assessment of parents' level of awareness or understanding of ADHD. Therefore, we are unable to provide specific data or analysis on parents' awareness or understanding of ADHD. However, we assume that parents participating in this study made judgments based on their observations of their children's behaviors and daily interactions when completing the questionnaire.

**Responses to suggestions for improvement:**

**Question 4**: Enhance the clarity of key concepts in the introduction, particularly regarding how different screen contents might influence ADHD risks differently.

**Answer:** Thank you for your valuable feedback on our manuscript. We have carefully considered your suggestion to enhance the clarity of key concepts in the introduction, particularly regarding how different screen contents might influence ADHD risks differently. Existing studies on the link between screen time and hyperactive behavior generally indicate that excessive screen time may indirectly increase the risk of ADHD by affecting children's sleep, physical activity, and social interaction[9, 10]. However, these studies often fail to explore the differential impacts of various types of screen contents on children's mental health in depth[11, 12]. For instance, educational videos, often perceived as beneficial for learning, may nevertheless contain rapidly changing visuals and intense sensory stimulation that adversely affect children's ability to concentrate and self-regulate. Similarly, cartoon videos, with their vibrant colors and exaggerated movements, may overstimulate children's attentional systems, leading to decreased attention to the real world and affecting cognitive and emotional development.[13]. In contrast, interactive videos, which promote children's active participation and social interaction, may have different effects on ADHD risk, though their specific impact requires further investigation.

**Question 5**:Improve the visualization of results, possibly through graphical summaries like forest plots for odds ratios.

**Answer:** Thank you for your valuable feedback on our manuscript.

Forest plot for odds ratios has been inserted as Fig 1.
