# Supplementary material for: The relationship between screen time, screen content for children aged 1-3, and the risk of ADHD in preschools
Source: PLoS One. 2025 Apr 23;20(4):e0312654. doi: 10.1371/journal.pone.0312654 (PMC12017831; doi:10.1371/journal.pone.0312654)
Supplement: Attachment — Submitted filename: Response to Reviewers (Second revision).docx [file pone.0312654.s005.docx]

**Reviewer 1:**

**Question 1:** Explicitly acknowledge potential selection bias due to parental self-reporting, particularly regarding screen time and ADHD symptoms.

**Answer:** Thank you for your suggestion. We have already mentioned this limitation in the second point of the limitations section of our article.

Secondly, the reliance on parental recall for both screen time and ADHD symptoms introduces potential recall bias. Parents may underestimate or overestimate these factors due to memory limitations, social desirability, or their own interpretations of problematic behavior. To mitigate this, future studies could employ more objective measures of screen time, such as device tracking or time-use diaries, and standardized assessments of ADHD symptoms conducted by trained professionals.

**Reviewer 2:**

First of all, we would like to express our sincere gratitude to the reviewer for the thorough review and valuable comments. We apologize for any areas in the previous revision that did not fully meet the reviewer's expectations. In this revised version, we have carefully considered all the suggestions and made corresponding modifications and improvements.

**Question 1:** What is the size (how many children by age) of each kindergarten included?

**Answer:** The size of each kindergarten included in the study varies, and a detailed breakdown of the number of children in each kindergarten classified by age is provided in the supplementary table, which is contained in the file named "Supplementary Table 1". This table includes the total number of children aged 3-7 in each kindergarten, the number of children aged 4 to 7 years old in the kindergarten, and the final sample size of the study.

**Question 2:** Are children obliged to attend kindergarten?

**Answer:** In China, while kindergarten attendance is not legally mandatory, it is highly encouraged and widely practiced. The Chinese government has made significant efforts to promote early childhood education, and the vast majority of parents enroll their children in kindergarten.

**Question 3:** What is the total population in the region covered, by age?

**Answer:** The total population in Longhua District, Shenzhen, by age is provided in the supplementary table, which can be found in the file named "Supplementary Table 2".

**Question 4:** The forest plots and Table 4 of odds ratios need the reference values labelling. Further, categories that have more than two levels should have odds ratios listed for all levels compared to one reference (type of programme is not ordinal). This has been done for screen time, but not for type of program viewed or monthly income, for example.

**Answer:** We are sincerely appreciated for your valuable comments. Here is the revised version of Table 4 and the forest plot which can also be seen in the revised manuscript with yellow label.

**Question 5:**

We appreciate your concerns regarding the interactive video group and the apparent discrepancy in the ADHD risk percentages. Below, we provide a detailed response to your questions and concerns.

Explanation of Interactive Video Group Findings

①High Percentage of ADHD Risk in Interactive Video Group:The observation that the interactive video group has the highest percentage of children at risk of ADHD (29.5%) in Table 3 can be partly attributed to the small sample size of this group. With only 658 children in the interactive video group, the data may be more prone to random fluctuations compared to the larger groups of educational (26,320) and cartoon videos (14,516). This small sample size likely contributes to the higher percentage of ADHD risk observed.

②Screen Time and ADHD Risk in Interactive Videos:

Our analysis in Table 5 shows that screen time does not appear to significantly affect the risk of ADHD in the interactive video group. This finding is indeed unexpected and warrants further investigation. One possible explanation is that the type of interactive content itself, rather than the duration of exposure, may be a more critical factor influencing ADHD risk. Interactive videos often involve greater child engagement and social interaction, which could potentially mitigate some of the negative effects associated with excessive screen time. We also give explanations in the discussion section. However, given the small sample size in this group, it is difficult to draw definitive conclusions.

③Crude OR Calculation:

You have correctly pointed out the crude OR calculation based on the frequencies in Table 3, which shows a higher OR for interactive videos compared to educational and cartoon videos. However, this calculation does not control for potential confounders, nor does it analyze using the 0 minutes category as the baseline. Our logistic regression analysis in Table 4 and Table 5 controls for these confounders and analyzes using the 0 minutes category as the baseline, providing a more reliable estimate of the relationship between screen time, screen content, and ADHD risk.

| **Category of screen time** | **No. of ADHD**  **risk children** | **No. of normal children** | **Total** |
| --- | --- | --- | --- |
| **0 mins/d** | **418** | **3,359** | **3,777** |
| **1~60 mins/d** | **5,667** | **29,276** | **34,943** |
| **61~120 mins/d** | **660** | **1,804** | **2,464** |
| **>120 mins/d** | **104** | **206** | **310** |
| **Total** | **6,849** | **34,645** | **41,494** |

**Question 6:** Typo in the introduction ‘howevre’ should read ‘however’

**Answer:** We sincerely thank for your reminding. ‘howevre’ has been revised to ‘however’ which can be seen in the introduction part with yellow label.
