# Supplementary material for: The relationship between screen time, screen content for children aged 1-3, and the risk of ADHD in preschools
Source: PLoS One. 2025 Apr 23;20(4):e0312654. doi: 10.1371/journal.pone.0312654 (PMC12017831; doi:10.1371/journal.pone.0312654)
Supplement: Attachment — Submitted filename: Response to Reviewers (Third revision).docx [file pone.0312654.s006.docx]

**Reviewer :**

Thank you for your suggestion. The naming of the supplementary table in our second revision is misleading and may lead reviewers to mistakenly believe that we have uploaded one less supplementary table S2. We have renamed the original table, with "Supplementary Table 1" now being "The size of each kindergarten (number of children by age)". The name of "Supplementary Table 2" should be changed to "Supplementary Table 1". In addition, we have anonymized the names of the kindergartens.

**Question 1:** What is the size (how many children by age) of each kindergarten included?

**Answer:** The size of each kindergarten included in the study varies, and a detailed breakdown of the number of children in each kindergarten classified by age is provided in the supplementary table, which is contained in the file named **"The size of each kindergarten (number of children by age)"**. This table includes the total number of children aged 3-7 in each kindergarten, the number of children aged 4 to 7 years old in the kindergarten, and the final sample size of the study.

**Question 3:** What is the total population in the region covered, by age?

**Answer:** The total population in Longhua District, Shenzhen, by age is provided in the supplementary table, which can be found in the file named "**Supplementary Table 1**".
